# PRMT1‐mediated arginine methylation promotes YAP activation and hepatocellular carcinoma proliferation

**DOI:** 10.1002/2211-5463.13909

**Published:** 2024-10-04

**Authors:** Jian Yu, Beibei Yu, Zushun Peng, Jianfeng Zhang, Juhui Sun, Bo Yang, Liushiyang Xu, De Luo

**Affiliations:** ^1^ Xiangshan First People's Hospital Medical and Health Group The Affiliated Xiangshan Hospital of Wenzhou Medical University Luzhou China; ^2^ Department of Hepatobiliary and Pancreatic Surgery The Affiliated Hospital of Southwest Medical University Luzhou China

**Keywords:** hepatocellular carcinoma, Hippo, PRMT1, YAP

## Abstract

The activity of Hippo signaling is commonly dysregulated in various human malignancies, including hepatocellular carcinoma (HCC). YAP, the key effector of Hippo pathway, is regulated through several posttranslational modifications. However, the mechanism by which YAP is regulated by arginine methylation remains unknown. In this study, immunoprecipitation and mass spectrometry were used to identify the arginine methylation site of YAP in HCC cells. The transcriptional activity of YAP and TEAD were further characterized by real‐time qPCR and immunofluorescence assay, and a subcutaneous and orthotopic tumor mouse model was used to assess the effect of PRMT1‐knockdown on HCC tumor growth. The result of mass spectrometry analysis identified that YAP was methylated at arginine 124. Moreover, we found that arginine methyltransferase PRMT1 interacted with YAP to mediate its arginine methylation, thus inhibited YAP phosphorylation and promoted YAP activity in the nucleus. PRMT1 was up‐regulated in HCC tissues and positively associated with the expressions of YAP target genes. Silencing PRMT1 in HCC cells inhibited cell proliferation and tumor growth, while PRMT1‐overexpression promoted HCC growth through YAP methylation. Our study reveals that PRMT1‐mediated arginine methylation at R124 is mutually exclusive with YAP S127 phosphorylation, thereby facilitating YAP activity in the nucleus and promoting tumorigenesis in HCC.

## Graphical abstract

PRMT1 facilitates the asymmetric di‐methylation of YAP at arginine 124, thereby diminishing the interaction between YAP and LATS1, inhibiting subsequent phosphorylation of YAP, and promoting both YAP activity and cell proliferation in hepatocellular carcinoma. Our study reveals a reciprocal crosstalk between methylation at the R124 site and phosphorylation at the S127 site of YAP, suggesting their mutually exclusive regulation.

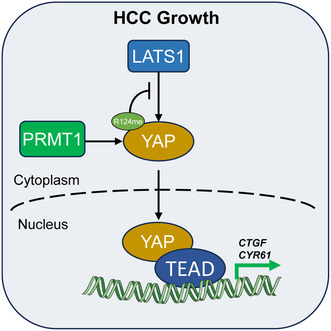

**Abbreviations**
ADMA: asymmetric di‐methyl arginine
BCL2: B‐cell leukemia/lymphoma‐2
BLI: Bioluminescence imaging
CRM1: chromosomal maintenance 1
CTGF: connective tissue growth factor
CYR61: cystein‐rich protein 61
DMEM: Dulbecco's modified eagle medium
FBS: fetal bovine serum
HCC: hepatocellular carcinoma
LATS1/2: large tumor suppressor 1/2
MST1: mammalian sterile 20‐like kinase 1
OGT: O‐GlcNAc transferase
PHGDH: phosphoglycerate dehydrogenase
PRMT1: protein arginine methyltransferase 1
qPCR: quantitative polymerase chain reaction
SDMA: symmetric di‐methyl arginine
SOX9: SRY‐box transcription factor 9
STAT3: signal transducer and activator of transcription 3
SUMO: small ubiquitin‐like modifier
TAZ: transcriptional co‐activator with a PDZ‐binding domain
TEAD: transcriptional enhanced associate domain
YAP: yes‐associated protein

Hepatocellular carcinoma (HCC) is a highly aggressive and deadly malignancy that poses a significant global health concern [1]. With a high mortality rate, HCC is often diagnosed at advanced stages, making it challenging to treat effectively [2, 3]. Clinical treatment for HCC involves a multidisciplinary approach that combines surgical interventions, such as liver resection or transplantation, with various other therapeutic modalities [2, 4, 5]. Despite advancements in liver cancer treatment, the overall prognosis remains poor, primarily due to late‐stage diagnosis and limited treatment options. Therefore, there is an urgent need for further research to identify novel therapeutic targets and develop more effective treatment strategies. Understanding the molecular mechanisms underlying the development and progression of HCC holds great promise in improving treatment outcomes and ultimately saving lives.

Hippo pathway is a highly conserved cellular signaling pathway that played vital roles in controlling organ size through regulating cell proliferation and apoptosis [6, 7, 8]. The dysregulation of Hippo signaling has garnered significant attention in recent years due to its involvement in various human cancers, including HCC [6, 9, 10]. When the Hippo pathway is intact, it restricts cell proliferation and promotes cell death, thereby preventing the formation of tumors. However, alterations of the components in this pathway can lead to its dysregulation, resulting in uncontrolled cell growth and the development of liver cancer [6, 11]. For instance, aberrant activation or inactivation of specific components within the pathway, such as the Hippo kinases (MST1/2) or their downstream effectors (YAP/TAZ), has been observed in HCC patients [11, 12, 13]. Furthermore, dysregulated Hippo signaling has been associated with poor prognosis and treatment resistance in liver cancer [12].

The activity of YAP, the key effector of Hippo pathway, is tightly regulated through posttranslational modifications, which determine its subcellular localization, stability, and interaction with TEAD transcriptional factors. These modifications include phosphorylation, acetylation, ubiquitination, methylation, and SUMOylation, among others [14, 15, 16, 17, 18]. Through these modifications, YAP undergoes dynamic changes in its activity and function, ultimately influencing cellular responses and contributing to disease development. Specifically, YAP is methylated by SET‐domain‐containing lysine methyltransferase Set7 at lysine 494 to promote its cytoplasmic retention [15]. Meanwhile, Set1A‐mediated mono‐methylation of YAP at K342 impedes its nuclear export by CRM1 and enhances its activity and tumorigenic potential [14]. Understanding the intricate network of YAP posttranslational modifications is essential for unraveling the underlying mechanisms of YAP signaling and may offer potential therapeutic targets for diseases such as liver cancer.

In this study, we found that YAP was di‐methylated at a conserved arginine residue. Arginine methyltransferase PRMT1 interacted with YAP to mediate its methylation. Depletion of PRMT1 in HCC cells inhibited the expressions of YAP target genes and cell proliferation. The expression of PRMT1 was up‐regulated in HCC tissues and correlated with poor prognosis of HCC patients. Overall, we discovered a novel regulatory mechanism of YAP activity in HCC, that may provide new insights into HCC clinical treatment.

## Materials and methods

### Cell culture and transfection

HepG2, Hep3B, and Hepa1‐6‐luciferase cells were obtained from the SunnCell company (Wuhan, China). Cells were maintained in DMEM culture medium (Gibco, Waltham, MA, USA) with 10% FBS (Biological Industries, Cromwell, CT, USA) and 1% penicillin/streptomycin (Gibco) at 37 °C in a humidified atmosphere containing 5% CO_2_. PRMT1 siRNA was designed and synthesized by the GenePharma Company (Shanghai, China) and transfected into cells with Lipofectamine 3000 (Thermo Fisher Scientific, Waltham, MA, USA). The sequences were siPRMT1‐1: CCGGCAGTACAAAGACTACAA, siPRMT1‐2: GTGTTCCAGTATCTCTGATTA.

### Data analysis of liver hepatocellular carcinoma (LIHC) patients

Correlations between PRMT1 and YAP‐TEAD target genes were analyzed using Timer2.0 database following the developer's instructions [19]. PRMT1 expression levels and prognosis of LIHC patients were analyzed using GEPIA database following the developer's instructions [20]. In brief, we inputted PRMT1 into the “Gene” field and selected LIHC in the “Dataset Selection” field, followed by clicking on “add” to construct a dataset list in the “Dataset” field. Subsequently, by clicking on the “Plot” button, GEPIA will generate a box plot illustrating the expression of PRMT1 in LIHC. The relationship between PRMT1 expression and the survival of LIHC patients was investigated using a similar analytical approach in GEPIA database.

### Western blot assay

Cells were lysed with RIPA lysis buffer (Beyotime Biotechnology, Nantong, Jiangsu, China) and separated by SDS/PAGE gel. The proteins were transferred to PVDF membrane (Millipore, Burlington, MA, USA) through a semi‐dry system (Bio‐Rad, Hercules, CA, USA) and then incubated with primary antibodies at 4 °C overnight. The images were scanned with a Chemiluminescent Imaging and Analysis System (Tanon, Shanghai, China). The antibodies used were: ADMA antibody (13522s; Cell Signaling Technology, Danvers, MA, USA), SDMA antibody (13222s; Cell Signaling Technology), LATS1 antibody (9153s; Cell Signaling Technology), Phospho‐LATS1‐Thr1079 antibody (8654s; Cell Signaling Technology), Phospho‐YAP antibody (13008s; Cell Signaling Technology), YAP antibody (sc‐101199; Santa Cruz, Dallas, TX, USA), Flag antibody (14793s; Cell Signaling Technology), HA antibody (3724s; Cell Signaling Technology), Actin antibody (AC038; ABclonal, Wuhan, Hubei, China), PRMT1 antibody (ab73246; Abcam, Thebarton, SA, USA), LAMIN antibody (10 298‐1‐AP; Proteintech, Wuhan, Hubei, China), GAPDH antibody (60004‐1‐Ig; Proteintech), HRP‐conjugated Goat anti‐Rabbit IgG (AS014; ABclonal), HRP‐conjugated Goat anti‐Mouse IgG (AS003; ABclonal).

### Immunofluorescence assay

Cells were seeded in confocal dishes and then fixed with 4% formaldehyde. After permeabilization with 0.2% Triton X‐100, the cells were blocked with 1% BSA in PBST buffer and subsequently incubated with primary antibodies at 4 °C overnight. After incubating with secondary antibodies, the cell images were viewed with a Zeiss LSM880 confocal microscope (Jena, Germany).

### Real‐time qPCR

Total RNA was extracted from cultured cells with RNA Isolater Extraction Reagent (Vazyme, Nanjing, Jiangsu, China) and then reverse transcribed to cDNA using HiScript II 1st Strand cDNA Synthesis Kit (Vazyme). Real‐time qPCR was performed using a QuantStudio 7 Flex Real‐time PCR system (Life Technologies, Waltham, MA, USA) with SYBR Green qPCR master mix (Yeasen, China). The primers used were: CYR61‐forward: GGTCAAAGTTACCGGGCAGT, reverse: GGAGGCATCGAATCCCAGC, CTGF‐forward: AAAAGTGCATCCGTACTCCCA, reverse: CCGTCGGTACATACTCCACAG, BCL2‐forward: GGTGGGGTCATGTGTGTGG, reverse: CGGTTCAGGTACTCAGTCATCC, GAPDH‐forward: GAAGGTGAAGGTCGGAG, reverse: GAAGATGGTGATGGGATTTC.

### Mouse model

Male nude mouse (6‐week‐old) were purchased from the Model Animal Research Center of Nanjing University (Nanjing, China). All animal care and handling procedures were performed in accordance with the National Institutes of Health Guide for the Care and Use of Laboratory Animals, and are approved by the Institutional Review Board of The Affiliated Xiangshan Hospital of Wenzhou Medical University (XYYJ‐2024‐631). Mice were housed in ventilated cages under specific pathogen‐free conditions with a 12 h light/dark cycle and *ad libitum* access to food and water. For mouse HCC subcutaneous tumor model, 5 × 10^6^ Hep3B‐luciferase cells were resuspended in 100 μL DMEM medium containing 10% matrigel and subcutaneously injected into the right flank of nude mice. The growth of tumors was monitored using an *in vivo* imaging system weekly. For mouse HCC orthotopic tumor model, Hepa1‐6‐luciferase cells (10^6^ cell⋅mouse^−1^) were orthotopically injected into the left lateral liver lobe of nude mice. The growth of liver tumors was assessed through palpation at least three times per week and visualized using an *in vivo* imaging system weekly. In the event of severe illness, a moribund condition or tumor size exceeding 20 mm in any direction during the experiment, we would implement humane endpoints for mice. All animals were alive until experimental end point in our study. Mice were sacrificed by asphyxiation using CO_2_ followed by bilateral thoracotomy at day 28 after tumor cell injection and livers were fixed for further analysis.

### Statistical analysis

All experiments were performed at least three times independently. The results were analyzed using GraphPad Prism 9 and shown as mean ± SD. Student's *t*‐test was employed to calculate the statistical significance of each difference. *P* < 0.05 was statistically significant.

## Results

### YAP is methylated at arginine 124

**Fig. 1:**
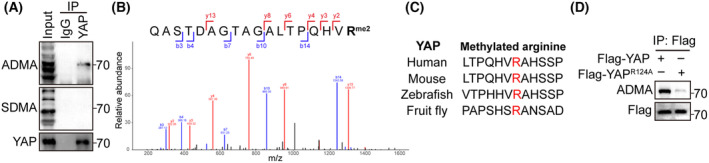
YAP is methylated at arginine 124. (A) Western blot analysis of immunoprecipitated YAP from HepG2 cells using ADMA and SDMA antibody. (B) Mass spectrum of YAP 107–124 peptide with di‐methylation at arginine 124. (C) Sequence alignment of YAP R124 from the indicated species. (D) Western blot analysis of wild‐type YAP and R124A mutant using ADMA antibody.

To explore whether YAP in HCC cells undergo arginine methylation, we examined immunoprecipitated endogenous YAP with anti‐symmetric di‐methyl arginine (SDMA) and asymmetric di‐methyl arginine (ADMA) antibody. The results showed that YAP was asymmetrically di‐methylated in HCC cells (Fig. 1A). We further determined the methylation site using mass spectrometry analysis and found that the arginine 124 (R), an evolutionarily conserved residue of YAP, was di‐methylated (Fig. 1B,C). Moreover, mutation of R124 completely abolished YAP methylation (Fig. 1D). Together, these data suggest that YAP is asymmetrically di‐methylated at R124 in HCC cells.

### PRMT1 interacts with YAP to mediate its arginine methylation

**Fig. 2:**
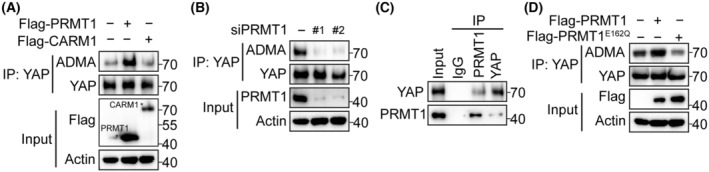
PRMT1 interacts with YAP to mediate its arginine methylation. (A) Western blot analysis of YAP methylation in PRMT1‐ and CARM1‐overexpressed HepG2 cells. (B) Western blot analysis of YAP methylation in PRMT1‐knockdown HepG2 cells. (C) Co‐immunoprecipitation analysis of the interaction between PRMT1 and YAP. (D) Western blot analysis of YAP methylation in wild‐type and E162Q PRMT1‐overexpressed HepG2 cells.

To identify the arginine methyltransferase that mediates YAP R124 methylation, we co‐transfected HepG2 cells with YAP and two main arginine asymmetric di‐methylation transferase, PRMT1 and CARM1, and found that PRMT1 significantly promoted YAP methylation at R124 (Fig. 2A). We next silenced PRMT1 in HepG2 and Hep3B cells and found that YAP methylation was significantly decreased (Fig. 2B; Fig. S1A). Notably, co‐immunoprecipitation assay confirmed the interaction between YAP and PRMT1 in HCC cells (Fig. 2C; Fig. S1A). Moreover, to determine whether YAP methylation is dependent on the catalytic activity of PRMT1, we mutated E162 to Q to generate a catalytic‐dead mutant of PRMT1. Co‐expression of PRMT1‐E162Q had no effect on YAP methylation compared to wild‐type PRMT1 (Fig. 2D). These data suggest that PRMT1 interacts with YAP to catalyze its methylation at R124.

### PRMT1 promotes YAP‐TEAD transcription activity

**Fig. 3:**
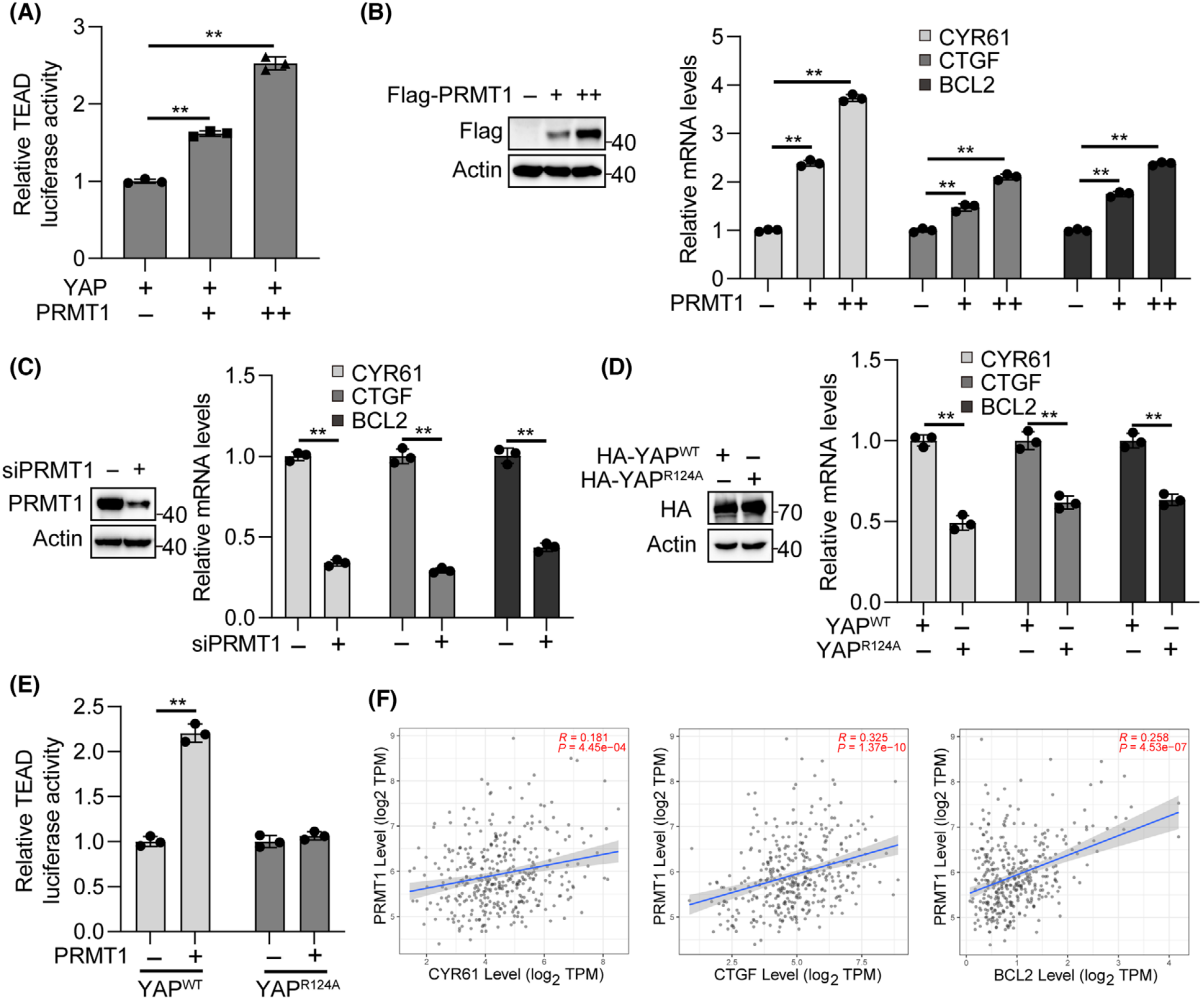
PRMT1 promotes YAP‐TEAD transcription activity. (A) TEAD luciferase activity in YAP‐ and PRMT1‐overexpressed HepG2 cells (*n* = 3). +1 μg, ++2 μg PRMT1 plasmids for 6 well plates. (B) Western blot analysis of PRMT1 levels in Flag‐PRMT1‐overexpressed HepG2 cells (left). Real‐time qPCR analysis of the expression levels of CTGF, CYR61 and BCL2 in PRMT1‐overexpressed HepG2 cells (right) (*n* = 3). +1 μg, ++2 μg PRMT1 plasmids for 6 well plates. (C) Western blot analysis of PRMT1 levels in PRMT1‐knockdown HepG2 cells (left). Real‐time qPCR analysis of the expression levels of CTGF, CYR61 and BCL2 in PRMT1‐knockdown HepG2 cells (right) (*n* = 3). (D) Western blot analysis of YAP levels in wild‐type‐ and R124A‐YAP‐overexpressed HepG2 cells (left). Real‐time qPCR analysis of the expression levels of CTGF, CYR61 and BCL2 in wild‐type‐ and R124A‐YAP‐overexpressed HepG2 cells (right) (*n* = 3). (E) TEAD luciferase activity in YAP wild‐type and R124A mutant co‐transfected with PRMT1 in HepG2 cells (*n* = 3). (F) Correlation analysis of the expression of *PRMT1* and YAP‐TEAD target genes in HCC tissues from Timer2.0 database. The data in A–E are presented as mean ± SD. Student's *t*‐test were used to determine statistical significance. **P < 0.01.

As a transcriptional co‐activator, YAP can promote target gene expressions through binding to TEADs transcription factor. We found that PRMT1‐overexpression significantly increased YAP‐mediated TEAD4 reporter activity (Fig. 3A) and the expression of YAP‐TEADs target genes *CTGF*, *CYR61*, and *BCL2* in a dose‐dependent manner (Fig. 3B; Fig. S2A). In contrast, PRMT1‐knockdown suppressed the expressions of these target genes in HCC cells (Fig. 3C; Fig. S2B). Moreover, we investigated the impact of YAP R124A mutant on the expression of YAP‐TEADs target genes and found that compared to R124A mutant, wild‐type YAP significantly activated expression of target genes (Fig. 3D; Fig. S2C). Next, we examined the role of R124 methylation in PRMT1‐mediated YAP activation and found that PRMT1‐overexpression failed to activate YAP‐R124A mutant compared to wild‐type YAP (Fig. 3E). Additionally, we performed a correlation analysis of PRMT1 and YAP‐TEADs target genes from Timer2.0 database and found that the expression of *PRMT1* was positively associated with the expressions of *CTGF*, *CYR61*, and *BCL2* in HCC tissues (Fig. 3F). These data together suggest that PRMT1 promotes YAP‐TEADs transcriptional activity in HCC.

### PRMT1 inhibits YAP phosphorylation and promotes YAP nuclear localization

**Fig. 4:**
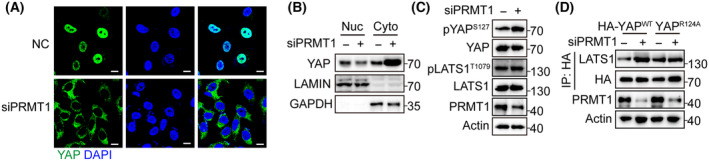
PRMT1 inhibits YAP phosphorylation and promotes YAP nuclear localization. (A) Immunofluorescence analysis of YAP cellular distribution in PRMT1‐knockdown HepG2 cells. Scale bar = 5 μm. (B) Nucleocytoplasmic separation analysis of YAP cellular distribution in PRMT1‐knockdown HepG2 cells. (C) Western blot analysis of LATS1, pLATS1^T1079^, YAP, and pYAP^S127^ levels in PRMT1‐knockdown Hep3B cells. (D) Co‐immunoprecipitation analysis of the interaction between LATS1 and wild‐type YAP or R124A YAP in PRMT1‐knockdown Hep3B cells.

As YAP nuclear localization is essential for its activation, we next examined the effect of PRMT1 on YAP cellular distribution in HCC cells. The result of immunofluorescence assay indicated that YAP was mainly localized in the cytosol in PRMT1‐knockdown HCC cells (Fig. 4A). Consistently, nucleocytoplasmic separation assay showed that PRMT1‐knockdown promoted YAP translocation from the nucleus to the cytoplasm (Fig. 4B). Chen et al. reported that PRMT1 inhibits LATS1 expression and subsequent YAP phosphorylation in chondrosarcoma development [21]. Thus, we next examined the impact of PRMT1 on YAP and upstream kinase LATS1 in HCC. However, our data indicated that PMRT1‐knockdown increased YAP S127 phosphorylation, but have no effect on LATS1 in Hep3B cells (Fig. 4C). In this regard, we next explored the effect of PRMT1 on the interaction between YAP and LATS1. Intriguingly, we observed that PRMT1 knockdown enhanced the interaction between LATS1 and wild‐type YAP, while no such effect was observed with the R124A mutant (Fig. 4D). These results suggest that PRMT1‐mediated YAP methylation impedes its interaction with LATS1 and phosphorylation, thus promoting its nuclear localization in HCC cells.

### PRMT1‐mediated YAP methylation promotes HCC tumor growth

**Fig. 5:**
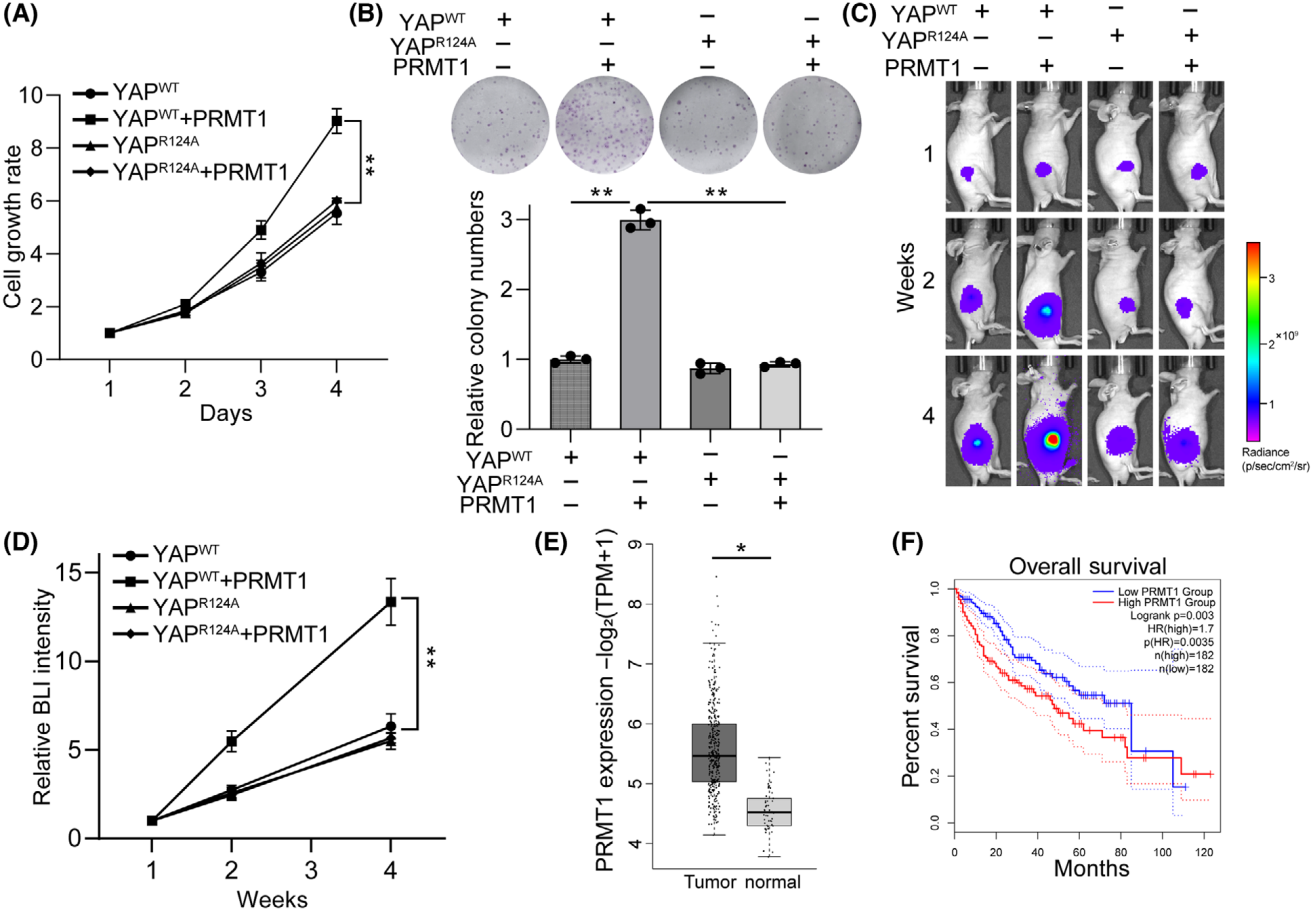
PRMT1‐mediated YAP methylation promotes HCC tumor growth. (A) CCK‐8 cell growth analysis of wild‐type or R124A YAP‐overexpressed Hep3B cells transfected with PRMT1 (*n* = 3). (B) Colony formation analysis of wild‐type or R124A YAP‐overexpressed Hep3B cells transfected with PRMT1 (*n* = 3). (C) Bioluminescence imaging (BLI) to show the tumor growth of nude mice injected with wild‐type or R124A YAP‐overexpressed Hep3B cells transfected with PRMT1. (D) Relative BLI intensity of tumor growth in C (*n* = 5). (E) PRMT1 expression levels in HCC tissues and adjacent tissues from GEPIA database (Tumor tissue number = 369, Normal tissue number = 160). (F) Kaplan Meier plotter survival analysis of PRMT1 expression in HCC tissues from GEPIA database. The data in A, B, D, and E are presented as mean ± SD. Student's *t*‐test were used to determine statistical significance. *P < 0.05, **P < 0.01.

We next explored the role of PRMT1 on HCC tumor growth via YAP methylation. We found that PRMT1‐knockdown inhibited HCC cell growth (Fig. S3A). Moreover, we generated a PRMT1‐knockdown Hepa1‐6‐luciferase mouse cell line and examined the effect of PRMT1 in an orthotopic tumor mouse model. The result showed that PRMT1‐knockdown significantly reduced HCC growth *in vivo* (Fig. S3B,C). To further investigate whether the effect of PRMT1 on HCC growth is dependent on YAP methylation, we transfected wild‐type YAP or R124A mutant‐overexpressed Hep3B cells with PRMT1. Our findings demonstrate that PRMT1 significantly promoted cell growth in wild‐type YAP‐overexpressed HCC cells, while exerting marginal impact on cancer cells overexpressing the R124A mutant (Fig. 5A,B). Similar results were also observed in the subcutaneous mouse tumor model (Fig. 5C,D). Moreover, the data from GEPIA database indicated that PRMT1 expression was elevated in HCC tissues compared to adjacent normal tissues (Fig. 5E). In addition, high PRMT1 expression was associated with poor prognosis of HCC patients (Fig. 5F). Together, these data indicate that PRMT1 promoted HCC tumor growth through YAP methylation *in vitro* and *in vivo*.

## Discussion

The Hippo signaling is a highly conserved pathway that plays a crucial role in regulating cell growth, proliferation, and apoptosis [12, 13]. It was firstly discovered in fruit flies and found to control organ size [13]. In mammals, the Hippo pathway is composed by a series of kinases and downstream effector proteins, transcriptional co‐activator YAP/TAZ and transcriptional factors TEADs family. YAP/TAZ translocate into nucleus and interact with TEADs to promote transcriptions of target genes that involved in cell proliferation and apoptosis [8]. The activities of YAP/TAZ were regulated by upstream kinase‐mediated phosphorylation [8]. Upon phosphorylation by LATS1/2, YAP/TAZ were sequestered in the cytoplasm through interaction with proteins 14‐3‐3 and subsequently undergo ubiquitin‐mediated proteasomal degradation [8]. YAP/TAZ were also regulated by other posttranslational modifications. For example, methyltransferase SET1A was reported to catalyze YAP methylation at K342, thus promoted YAP activity via blocking CRM1‐mediated nuclear export of YAP. SET1A‐mediated YAP‐K342 methylation promoted cell proliferation and tumorigenesis [14]. Moreover, YAP is O‐GlcNAcylated by O‐GlcNAc transferase (OGT) at serine 109. OGT regulates YAP localization and activation, and played a role in tumorigenesis [16, 22]. Here, we reported PRMT1 as a regulator of Hippo‐YAP activity in liver cancer. PRMT1 interacted with YAP to catalyze its asymmetric di‐methylation and promoted YAP activity. Recently, Qian et al. also reported that PRMT1‐mediated methylation of YAP at residue R124 enhances its interaction with SOX9 and facilitates its translocation into the nucleus [23]. Consistently, we observed that PRMT1 catalyzes the methylation of YAP at R124, disrupting its interaction with the upstream kinase LATS1 and consequently suppressing YAP phosphorylation while promoting nuclear translocation in HCC cells.

Abnormalities in the Hippo pathway have been implicated in various types of cancer, including liver cancer. Dysregulation of this pathway can lead to uncontrolled cell growth and tumor formation. Understanding the Hippo pathway and its role in cancer has opened up new possibilities for targeted therapies. PRMT1 is an enzyme involved in the process of protein arginine methylation and play essential roles in various physiological and pathological processes, including human cancer [24, 25]. Previous studies have reported that PRMT1 promoted HCC cell growth and migration through regulating PHGDH activity and STAT3 signaling pathway [26, 27]. Similarly, we found that the expression of PRMT1 was up‐regulated in HCC tissues and correlated with poor prognosis of HCC patients. Our study showed that PRMT1 regulated YAP activity in HCC cells through arginine methylation. Moreover, our data indicated that the oncogenic role of PRMT1 in HCC growth is dependent on YAP methylation at arginine 124.

Recently, increasing studies have been focused on the regulation of Hippo‐YAP signaling in human cancer, however, its regulation by PRMT1‐mediated arginine methylation in HCC has not been reported yet. In the current study, we revealed that YAP was asymmetrically di‐methylated at arginine 124 by PRMT1. Silencing of PRMT1 reduced the expressions of Hippo‐YAP target genes in HCC cells. The activity of YAP was regulated by the upstream kinase LATS1/2 within the Hippo pathway. The LATS1/2 kinase has been reported to recognize the HXRXXS motif of YAP, thereby facilitating its phosphorylation and degradation [28]. Chen et al. reported that PRMT1 inhibits LATS1 expression and subsequent YAP phosphorylation in chondrosarcoma development [21]. However, our data indicated that PRMT1 did not affect LATS1 phosphorylation levels in HCC cells; instead, PRMT1‐mediated YAP methylation impaired the interaction between YAP and LATS1 and subsequent YAP S127 phosphorylation. A novel finding of our study is the identification of a reciprocal crosstalk between methylation at the R124 site and phosphorylation at the S127 site of YAP, suggesting their mutually exclusive regulation. Additionally, we found that PRMT1 was up‐regulated in HCC tissues compared to adjacent healthy tissues and correlated with the expressions of YAP downstream target genes. PRMT1‐depletion inhibited HCC cell proliferation and tumor growth. On the contrary, PRMT1‐overexpression promoted HCC growth in a YAP methylation‐dependent manner. In summary, our findings reveal novel molecular mechanisms underlying the regulation of YAP activity in HCC progression and may provide new insights into clinical treatment strategies for HCC.

## Conflict of interest

The authors declare that they have no conflict of interest.

## Author contributions

JY, BY and ZP performed most of the experiments. JY, JZ, LX and JS analyzed the data. JY, JS and BY wrote the manuscript. DL supervised the project.

## Supporting information


**Fig. S1.** PRMT1 interacts with YAP to mediate its arginine methylation.
**Fig. S2.** PRMT1 promotes YAP‐TEAD transcription activity.
**Fig. S3.** PRMT1‐mediated YAP methylation promotes HCC tumor growth.

## Data Availability

The data that support the findings of this study are available from the corresponding author upon reasonable request.
