# Supplementary material for: PRMT1‐mediated arginine methylation promotes YAP activation and hepatocellular carcinoma proliferation
Source: FEBS Open Bio. 2024 Oct 4;14(12):2104–12. doi: 10.1002/2211-5463.13909 (PMC11609590; doi:10.1002/2211-5463.13909)
Supplement: Supplementary file 1 — Fig. S1. PRMT1 interacts with YAP to mediate its arginine methylation. Fig. S2. PRMT1 promotes YAP‐TEAD transcription activity. Fig. S3. PRMT1‐mediated YAP methylation promotes HCC tumor growth. [file FEB4-14-2104-s001.docx]

**Supplemental Figures**


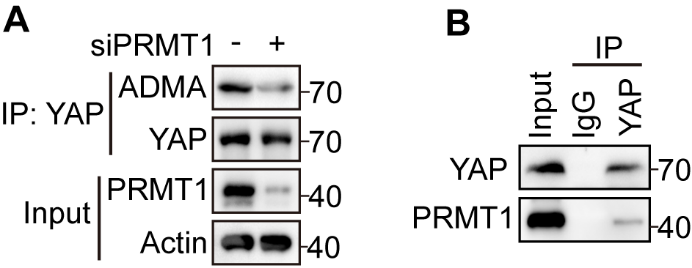


**Supplemental Figure 1. PRMT1 interacts with YAP to mediate its arginine methylation**

**A.** Western blot analysis of YAP methylation in PRMT1-knockdown Hep3B cells. **B.** Coimmunoprecipitation analysis of the interaction between PRMT1 and YAP in Hep3B cells.


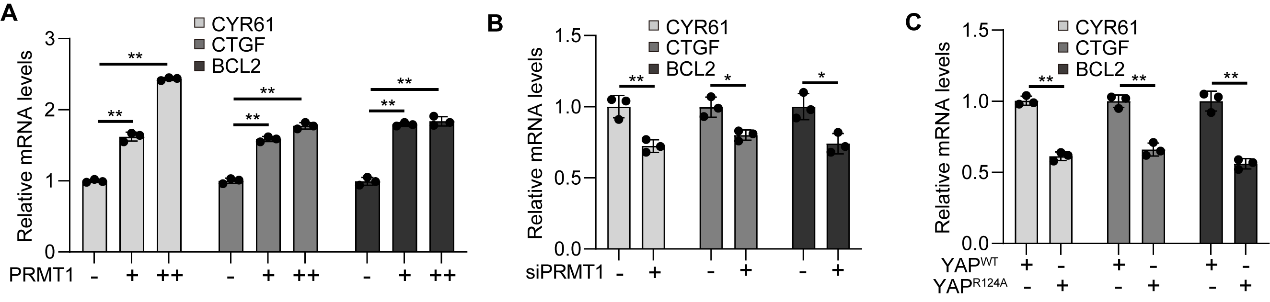


**Supplemental Figure 2. PRMT1 promotes YAP-TEAD transcription activity**

**A.** Real-time qPCR analysis of the expression levels of CTGF, CYR61 and BCL2 in PRMT1-overexpressed Hep3B cells. + 1ug, ++ 2ug PRMT1 plasmids for 6 well plates. **B.** Real-time qPCR analysis of the expression levels of CTGF, CYR61 and BCL2 in PRMT1-knockdown Hep3B cells. **C.** Real-time qPCR analysis of the expression levels of CTGF, CYR61 and BCL2 in wildtype- and R124A-YAP-overexpressed Hep3B cells. The data in A-C are presented as mean ± S.D. Student’s *t*-test were used to determine statistical significance. * p<0.05, ** p<0.01.


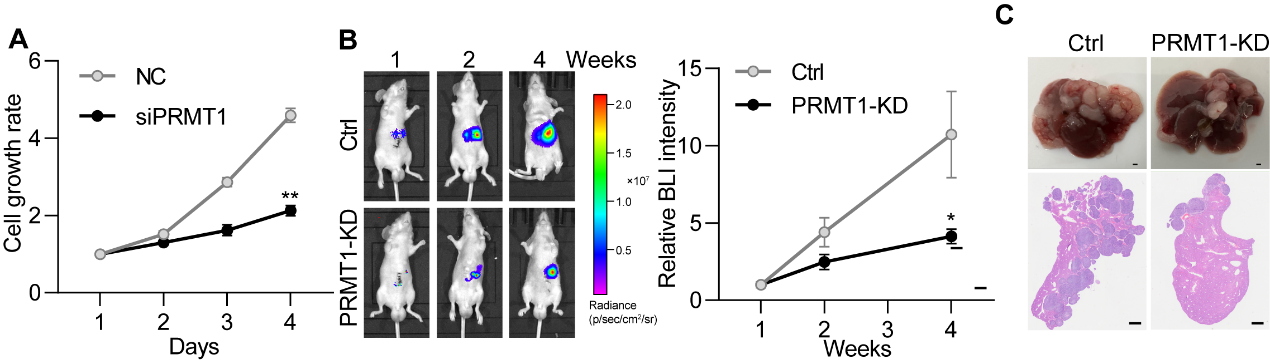


**Supplemental Figure 3. PRMT1-mediated YAP methylation promotes HCC tumor growth**

**A.** Cell growth analysis of PRMT1-knockdown HepG2 cells. **B, C.** BLI imaging (B) and HE staining (C) to show the tumor growth of PRMT1-knockdown Hepa1-6-luciferase cells in an orthotopic tumor mouse model (n = 5). Scale bar = 1 mm. The data in A and B are presented as mean ± S.D. Student’s *t*-test were used to determine statistical significance. * p<0.05, ** p<0.01.
